# Metabeliefs about worry, cognitive fusion, and acceptance: associations and mediations analysis

**DOI:** 10.3389/fpsyg.2025.1639105

**Published:** 2025-08-20

**Authors:** Francisco Sanchez-Escamilla, Marta Redondo-Delgado, Antonia María Jímenez-Ros, Miguel Ángel Pérez-Nieto

**Affiliations:** ^1^HM Faculty of Health Sciences, Camilo José Cela University, Madrid, Spain; ^2^HM Hospitals Health Research Institute, Madrid, Spain; ^3^University Center of Research in Psychology (CUIP), Faro, Portugal; ^4^Universidade do Algarve, Faro, Portugal

**Keywords:** anxiety, metacognition, acceptance, worry, mediation

## Abstract

**Background:**

Although metacognitive therapy (MCT) and acceptance and commitment therapy (ACT) are grounded in different theoretical frameworks, both target repetitive negative thinking (RNT) processes, such as worry and rumination, and share a focus on fostering psychological flexibility and reducing experiential avoidance. However, no integrated theoretical model currently exists to combine their potential strengths. Recent research highlights the importance of metacognitive beliefs and acceptance-related processes in maintaining maladaptive RNT.

**Objective:**

This study aimed to explore metacognitive and acceptance-based variables in relation to RNT.

**Methods:**

The sample consisted of 149 people (116 females), aged between 18 and 71 (M = 34.7; SD = 14.9) who answered the following questionnaires: PSWQ, The AAQ-II, The CFQ, The VQ, MCQ-30 and TCQ.

**Results:**

The mediation analysis reveals that cognitive fusion significantly mediated the relationship between metacognitive beliefs and worry, whereas acceptance had a weaker mediating effect.

**Conclusion:**

Beliefs about the uncontrollability of worry appear to play a pivotal role in sustaining worry, primarily through their influence on cognitive fusion. These findings provide preliminary support for conceptual overlaps between MCT and ACT in addressing RNT. However, as this is an exploratory and cross-sectional study, conclusions about treatment mechanisms should be drawn cautiously, and future longitudinal and experimental studies are needed to strengthen these insights.

## Introduction

Anxiety is one of the more common emotions people experience and which causes them the greatest distress, affecting as many as 30% of the global population (APA, 2024). A core feature of anxiety disorders is the presence of repetitive negative thinking (RNT), which encompasses processes such as worry and rumination (Ehring and Watkins, 2008). While worry refers to a future-oriented, repetitive cognitive activity focused on anticipated threats (Borkovec, 1994), whereas rumination involves dwelling on past events or perceived failures in a passive, often self-critical cycle (Nolen-Hoeksema et al., 2008). While both processes are forms of RNT and share overlapping features, they are conceptually distinct and may operate through different cognitive mechanisms. Intrusive thoughts, although similarly distressing and often involuntary, also differ from RNT because they are typically brief and episodic rather than sustained and elaborative (Clark, 2005; Rachman, 1997). This conceptual distinction is crucial for the present study, which focuses specifically on worry given its strong links to generalized anxiety and its central role in metacognitive models of pathological thought (Wells, 2009).

Both worry and intrusive thoughts have traditionally been treated through cognitive behavioral therapy (CBT) (Masuda et al., 2004). The emergence of a metacognitive and contextual approach in recent years has led to the intro-duction of interventions that have proven to be effective where traditional CBT has fallen short (Hayes and Wilson, 1994; Hayes, 2004).

These interventions include Metacognitive Therapy (MCT) and Acceptance and Commitment Therapy (ACT), which have proven to be particularly effective in treating a series of anxiety-related issues (Sánchez Escamilla et al., 2024; Wells, 2009; Hayes et al., 1999). Worrying is often associated with uncertainty over anticipated threats and a perceived lack of control over future events (Nolen-Hoeksema et al., 2008), though other factors may also contribute in certain contexts. Given the role of worry across various diagnoses (Harvey et al., 2004; Ehring and Watkins, 2008), several therapeutic approaches have been developed to target and reduce repetitive negative thinking (RNT) (Wells, 2009; Watkins, 2016). Such thoughts may be conceptualized as experiential avoidance—defined as attempts to evade or suppress unpleasant internal experiences (emotions, thoughts, bodily sensations)—which is a key focus of ACT (Hayes et al., 1999; Wilson and Luciano, 2002).

The metacognitive theory (Wells and Matthews, 1994; Wells, 2009) is based on the principle that negative thoughts and emotions are, for the most part, transient experiences. These experiences become persistent and evolve into psychological disorders when the individual adopts a particular thinking style that disrupts self-regulation and impedes resolution of stress-related content. MCT, grounded in the Self-Regulatory Executive Function (S-REF) model (Wells and Matthews, 2016), posits that metacognitive beliefs play a critical role in the activation and maintenance of RNT. Specifically, positive beliefs about worry (e.g., “worrying helps me cope”) and negative beliefs about uncontrollability and danger (e.g., “I cannot control my worry”) contribute to a Cognitive Attentional Syndrome (CAS) characterized by persistent worry, rumination, and maladaptive coping behaviors (Wells, 2009). A meta-analysis by Normann and Morina (2018) confirmed the efficacy of MCT for both anxiety and depression. Their findings suggest that MCT not only reduces targeted symptoms but also alleviates comorbid problems, supporting its role as a transdiagnostic intervention (Sun et al., 2017). These comorbid symptoms include anxious, uncontrollable, and excessive worrying (Meyer et al., 1990) and depressive rumination (Nolen-Hoeksema and Morrow, 1991), which underpin emotional problems (Papageorgiou and Wells, 2003). Recent studies have highlighted that metacognitive beliefs about negative emotions and cognitions are central to sustaining maladaptive RNT (Xu and Tsai, 2025; Kornacka et al., 2025).

From a metacognitive perspective, rumination is sustained by beliefs about its perceived utility and controllability. Positive metacognitive beliefs (e.g., “ruminating helps me to solve problems”) motivate engagement in this process, while negative metacognitive beliefs (e.g., “my rumination is uncontrollable and harmful”) exacerbate distress and reinforce avoidance strategies (Wells and Matthews, 2016; Papageorgiou and Wells, 2003). The S-REF model conceptualizes this as part of the Cognitive Attentional Syndrome (CAS), where dysfunctional metacognitive beliefs guide attention and coping responses that maintain repetitive negative thinking.

Recently, the HExAGoN model (Tamm et al., 2024) has been proposed as a comprehensive framework for understanding repetitive negative thinking (RNT), including both worry and rumination. This model emphasizes multiple interacting pathways—such as habit formation, executive control deficits, abstract processing, goal discrepancies, and negative cognitive biases—that contribute to the onset and maintenance of RNT. Importantly, HExAGoN integrates key elements from metacognitive theories, such as the role of positive and negative beliefs about cognition (Wells and Matthews, 2016), and self-regulatory processes, such as effortful control and goal management. This aligns conceptually with MCT’s Cognitive Attentional Syndrome (CAS), where metacognitive beliefs sustain maladaptive thinking, and with ACT’s focus on psychological inflexibility, where cognitive fusion and experiential avoidance perpetuate distress. By highlighting these overlapping mechanisms, the HExAGoN model offers a useful lens for exploring potential integration between MCT and ACT approaches in targeting RNT.

ACT, in contrast, is based on the psychological flexibility model and draws from Relational Frame Theory (RFT; Hayes et al., 2001). It targets processes such as experiential avoidance and cognitive fusion—rigid patterns of responding to internal experiences—through interventions designed to foster acceptance, defusion, and values-based action (Hayes et al., 2012). Cognitive fusion, in particular, refers to the tendency to become entangled with thoughts such that they dominate behavior, while experiential avoidance involves attempts to escape or suppress unwanted internal experiences, paradoxically amplifying distress over time (Hayes et al., 1996). ACT focuses on everything involved in an individual’s learning history, and repetitive negative thinking tends to become the pre-dominant reaction, thereby reducing short-term emotional distress, but prolonging it both in duration and intensity over the long term (Côté et al., 2002).

From the perspective of ACT, while psychological problems may originate from a general absence of relational skills (e.g., in cognitive deficits), a primary source of psychopathology is how language and cognition interact with direct contingencies. These interactions produce a reduced ability to persist or shift behavior in pursuit of long-term goals. ACT proposes that psychological inflexibility arises from weak or ineffective contextual control over language processes (Ong et al., 2024).

In contexts that promote fusion, human behavior is more influenced by rigid verbal networks than by direct environmental contingencies. This leads individuals to act in ways that are inconsistent with environmental opportunities and chosen values. Justificatory contexts place excessive emphasis on the internal causes of behavior—especially private, conditioned events—while experiential control contexts aim to eliminate or manage emotional and cognitive states, often using them as the sole metric for a successful life (Addis and Jacobson, 1996; Zettle and Hayes, 1986). These contexts are intertwined and help explain why cognitive fusion supports experiential avoidance: efforts to control private experiences often increase their functional salience and reduce behavioral repertoire (Hayes et al., 1996). Unfortunately, such avoidance tends to increase the prominence of aversive experiences and limits flexibility, as many valued behaviors may evoke precisely the private events one is trying to control.

Both MCT and ACT have been explored in numerous studies (see recent reviews: Nordahl et al., 2022; Sánchez Escamilla et al., 2024). Although MCT and ACT are rooted in distinct theoretical frameworks, recent evidence suggests potential overlap in their targeting of metacognitive processes and RNT (Sánchez Escamilla et al., 2024). Both approaches emphasize shifting the individual’s relationship to their thoughts—MCT by altering metacognitive beliefs and ACT by promoting cognitive defusion and acceptance. This convergence raises the possibility of shared mechanisms that could inform integrated treatment models (Nordahl et al., 2022).

The present study aims to contribute to this integration by examining whether cognitive fusion and experiential avoidance (key ACT processes) mediate the relationship between metacognitive processes (central to MCT) and worry. This focus on worry, rather than on other forms of RNT such as rumination, reflects its theoretical and clinical relevance in generalized anxiety and its prominent role in metacognitive models. Although rumination and worry share features of perseverance cognition, worry’s future-oriented, threat-related content aligns more directly with the mechanisms targeted by MCT and ACT. Specifically, we hypothesize that broader metacognitive factors, including beliefs about the controllability, usefulness, and monitoring of thoughts, will predict worry indirectly via these mediators. Such findings would provide preliminary evidence for common processes across MCT and ACT, informing future development of transdiagnostic interventions for RNT.

## Methods

### Procedure

The research protocol was approved by the Ethics Committee on Clinical Research (CEIC) at Camilo José Cela University (code: 13_23_AICAT).

The study was conducted in January 2025. Participants were not offered any form of reimbursement. Recruitment was carried out through two main strategies: first, university students from Camilo Jose Cela University were invited to participate voluntarily via an institutional message; second, a snowball sampling method was used by distributing a Google Forms link through social media platforms to reach a broader population.

The sample answered this battery of questionnaires in around 30 min.

### Participants

The sample consisted of 149 participants completed the evaluation, of whom 116 (77.9%) were female and 33 (22.1%) males, with an average age of 34.7 (±14.9 SD) who freely agreed to take part in the study. Information was also collected on the various questionnaires administered (see Table 1). Each one of them was as-signed a numerical code to ensure their anonymity and the confidentiality of their data. The inclusion criterion was a willingness to take part, while the exclusion criteria involved being under legal age or having a cognitive deficit.

**Table 1:** Demographic data.

Variables	Sub-variables	Total (*n* = 149)	(Min value – max value)	Skew	Kurtosis
Age M (±SD)		34.7 (±14.9)	18–71	0.689	−0.822
Sex *n* (%)					
	Female	116 (77.9%)			
	Male	33 (22.1%)			
Nationality *n* (%)	Spain	142 (95.3%)			
	Other	7 (4.7%)			
PSWQ M (SD)		42.6 (±8.96)	24–64	0.196	−0.625
AAQ-II M (SD)		20.5 (±9.31)	7–46	0.510	−0.541
CFQ M (SD)		24.8 (±10.2)	7–49	0.158	−0.906
VQ M (SD)					
	Obstruction	16.2 (±5.94)	5–32	0.171	−0.572
	Progress	25.6 (±4.74)	10–35	−0.245	0.118
MCQ-30 M (SD)					
	Beliefs	10.5 (±3.73)	6–22	0.850	0.325
	Uncontrollability	12.5 (±3.79)	6–22	0.460	−0.432
	Cognitive confidence	11.9 (±5.13)	6–24	0.773	−0.428
	Control of thoughts	11.9 (±4.07)	6–23	0.536	−0.171
	Self-consciousness	15.1 (±3.73)	7–24	0.255	−0.401
TCQ M (SD)					
	Distraction	15.7 (±3.02)	9–23	0.351	−0.165
	Reappraisal	14.9 (±3.07)	7–24	−0.014	−0.155
	Worry	12.3 (±3.15)	6–21	0.543	0.026
	Punishment	9.9 (±3.39)	6–20	0.977	0.209
	Social construct	11.7 (±3.13)	5–20	0.240	0.127

M, Media; SD, Standar desviation; AAQ-II, The Acceptance and Action Questionnaire – version 2; CFQ, The Cognitive Fusion Questionnaire; VQ, The Valuing Questionnaire; MCQ-30, Metacognitions Questionnaire-30; TCQ, Thought Control Questionnaire.

To assess potential heterogeneity resulting from the two recruitment methods (university students and snowball sampling), we compared key demographic and psychological variables between groups. Independent samples t-tests revealed significant differences in age (t(147) = −8.229, *p* < 0.001, d = 1.45) and several psychological measures, such as GAD-7 and AAQ-II (*p* < 0.05). A chi-square test also indicated a significant difference in gender distribution between groups (χ^2^(1) = 5.27, *p* = 0.022). However, no significant differences were found in the primary outcome variable (worry; PSWQ: t(147) = 0.786, *p* = 0.434, d = 0.13). These findings suggest some demographic and psychological variability across subgroups. Given that the primary outcome was unaffected, we proceeded with analyses on the combined sample while acknowledging this variability as a limitation.

### Instruments

The following questionnaires were administered for evaluation purposes:

The first step involved a questionnaire specifically developed for this study to gather each patient’s personal data, including sociodemographic variables such as sex, age, and nationality.

Worry was measured through the Penn State Worry Questionnaire (PSWQ) (Meyer et al., 1990; Sandín et al., 2009). It is a measure of the anxiety trait designed to appraise the general tendency to experience worry. It has 16 items that participants respond to using a five-point Likert scale that ranges from 1 (not at all typical of me) to 5 (very typical of me). The Spanish version by Sandín and Chorot (1995). It has a Cronbach’s alpha of 0.95 (Sandín et al., 2009).

The following questionnaires were used to measure psychological processes based on ACT:

- The Acceptance and Action Questionnaire – version 2 (AAQ-II) (Bond et al., 2011; Ruiz et al., 2013). It is a seven-item measure of psychological flexibility. Participants respond to the items using a seven-point Likert scale from 1 (not at all true) to 7 (completely true). It has a Cronbach’s alpha of 0.91. The scores in the tests using the AAQ-II have recorded good internal consistency and test–retest reliability in community samples (Ruiz et al., 2013).
- The Cognitive Fusion Questionnaire (CFQ) (Gillanders et al., 2014; Romero-Moreno et al., 2014). It consists of seven items that are responded to using a Likert scale with seven options (1 = Never true, 7 = Always true). A Spanish translation of the instrument was used (Romero-Moreno et al., 2014) in a version modified by Ruiz et al. (2017), whose study with a Columbian population recorded high alpha coefficients (between 0.89 and 0.93).
- The Valuing Questionnaire (VQ) (Smout et al., 2014; Ruiz et al., 2022). It measures values through two subscales: Obstruction (items: 1, 2, 6, 8, and 10) and Progress (items: 3, 4, 5, 7, and 9). Cronbach’s alpha values for the Progress subscale ranged from 0.81 to 0.85, yielding an overall alpha of 0.83. For the Obstruction subscale, Cronbach’s alphas ranged from 0.78 to 0.84, with an overall alpha of 0.82 (Ruiz et al., 2022).

The following questionnaires are used to measure psychological processes based on metacognitive therapy:

- Metacognitions questionnaire-30 (MCQ-30) (Wells and Cartwright-Hatton, 2004; Ramos-Cejudo et al., 2013). It assesses individual differences in metacognitive beliefs, judgments, and monitoring tendencies. It consists of five subscales involving 30 items. The responses to each item are scored on a four-point Likert scale, from 1 (I do not agree) to 4 (I totally agree). The five subscales measure the following dimensions: (1) positive beliefs about, (2) negative beliefs about uncontrollability and danger, (3) cognitive confidence, (4) need to control thoughts and (5) cognitive self-consciousness – awareness. It has a Cronbach’s alpha of 0.93 (Wells and Cartwright-Hatton, 2004).
- The Thought Control Questionnaire (TCQ) (Wells and Davies, 1994; Lucero, 2002). It was designed to understand the strategies that individuals use to control their thoughts and consists of 30 items, with the responses using a four-point scale. The Spanish version of TCQ showed an adequate internal consistency (Cronbach’s alpha for the total scale = 0.84) and convergent validity (Lucero, 2002).

In the present sample, internal consistency indices were calculated for all instruments. Cronbach’s alpha coefficients were as follows: PSWQ = 0.80, AAQ-II = 0.92, CFQ = 0.95, VQ–Obstruction = 0.87, VQ–Progress = 0.81, MCQ-30 total = 0.91, and TCQ total = 0.78. These results indicate acceptable to excellent internal reliability across all scales used in the study.

### Data analysis

The first step involved descriptive analyses for calculating each variable’s mean and standard deviation. This was followed by conducting a Pearson’s correlation coefficient for all the variables. Finally, mediation models were developed using MCQ-30 subscales and other variables that showed the strongest and most significant associations with worry in preliminary analyses. The final model retained was the one that demonstrated the strongest predictive relationships within the sample.

All the analyses were performed with Jamovi 2.3.28.

## Results

### Correlations among tests

The correlation analysis revealed that worry (PSWQ) correlates positively and significantly with cognitive fusion (CFQ; *r* = 0.72, *p* < 0.001) and uncontrollability (MCQ30 Uncontrollability; *r* = 0.64, *p* < 0.001).

Furthermore, AAQ-II correlates negatively with worry (PSWQ; *r* = −0.61, *p* < 0.001). Acceptance also correlates negatively with the obstruction of values (VQ Obstruction; *r* = −0.47, *p* < 0.001) and positively with progress in values (VQ Progress; *r* = 0.50, *p* < 0.001). In turn, beliefs about uncontrollability correlate positively with cognitive fusion (*r* = 0.74, *p* < 0.001) (Table 2).

**Table 2:** Correlation matrix.

	PSWQ		AAQII		CFQ		VQ obstruction		VQ progress		MCQ30 beliefs		MCQ30 uncontrollability		MCQ30 cognitive confidence		MCQ30 thought control		MCQ30 Self-consciousness		TCQ distraction		TCQ reappraisal		TCQ worry		TCQ punishment		TCQ social construct	
PSWQ	—																													
AAQII	−0.61	***	—																											
CFQ	0.72	***	0.86	***	—																									
VQ Obstruction	0.50	***	−0.47	***	0.71	***	—																							
VQ Progress	−0.18	*	0.50	***	−0.33	***	−0.54	***	—																					
MCQ30 Beliefs	0.43	***	0.37	***	0.4	***	0.20	*	−0.05		—																			
MCQ30 Uncontrollability	0.64	***	0.67	***	0.74	***	0.56	***	−0.34	***	0.35	***	—																	
MCQ30 Cognitive Confidence	0.29	***	0.30	***	0.28	***	0.39	***	−0.27	***	0.17	*	0.39	***	—															
MCQ30 Thought control	0.39	***	0.49	***	0.53	***	0.44	***	−0.31	***	0.5	***	0.63	***	0.39	***	—													
MCQ30 Self-consciousness	0.46	***	0.34	***	0.51	***	0.27	***	0.1		0.48	***	0.41	***	0.16	*	0.44	***	—											
TCQ Distraction	−0.15		−0.19	*	−0.16	*	−0.2	*	0.31	***	0.11		−0.16		−0.13		−0.04		0.05		—									
TCQ Reappraisal	0.32	***	0.25	**	0.36	***	0.21	*	0.08		0.39	***	0.28	***	0.13		0.23	**	0.6	***	0.19	*	—							
TCQ Worry	0.66	***	0.487	***	0.61	***	0.45	***	−0.19	*	0.46	***	0.62	***	0.22	**	0.48	***	0.52	***	−0.04		0.4	***	—					
TCQ Punishment	0.53	***	0.598	***	0.642	***	0.481	***	−0.35	***	0.43	***	0.642	***	0.282	***	0.61	***	0.33	***	−0.05		0.29	***	0.61	***	—			
TCQ Social Construct	0.06		−0.08		−0.07		−0.07		0.11		0.03		−0.05		−0.04		−0.17	*	0.09		−0.08		0.16		0.03		−0.14		—	

*p < 0.05; **p < 0.01; ***p < 0.001. AAQ-II, The Acceptance and Action Questionnaire – version 2; CFQ, The Cognitive Fusion Questionnaire; VQ, The Valuing Questionnaire; MCQ-30, Metacognitions Questionnaire-30; TCQ, Thought Control Questionnaire.

### Mediation model of worry

Data were analyzed using Jamovi (Version 2.3.28). Mediation analyses were performed using bias-corrected bootstrapping with 1,000 resamples and a 95% confidence interval. No covariates were included in the model, as the aim was to explore unadjusted relationships between metacognitive processes, cognitive fusion, acceptance, and worry in a non-clinical sample.

All assumptions for linear regression were examined. Linearity was assessed via scatter plots of each predictor against the outcome variable (PSWQ), showing approximately linear relationships. Normality of residuals was evaluated using a Q-Q plot and the Shapiro–Wilk test (W = 0.994, *p* = 0.792), with no significant deviations from normality observed. Homoscedasticity was confirmed by inspecting residuals versus fitted values plots, which displayed random distribution of residuals around zero. Multicollinearity was assessed using Variance Inflation Factor (VIF), with all VIF values below 5 (range: 1.20–4.70), indicating no serious multicollinearity issues. Multicollinearity was assessed using Variance Inflation Factor (VIF), with all VIF values below 5 (range: 1.20–4.70), indicating no serious multicollinearity issues.

**Figure 1:**
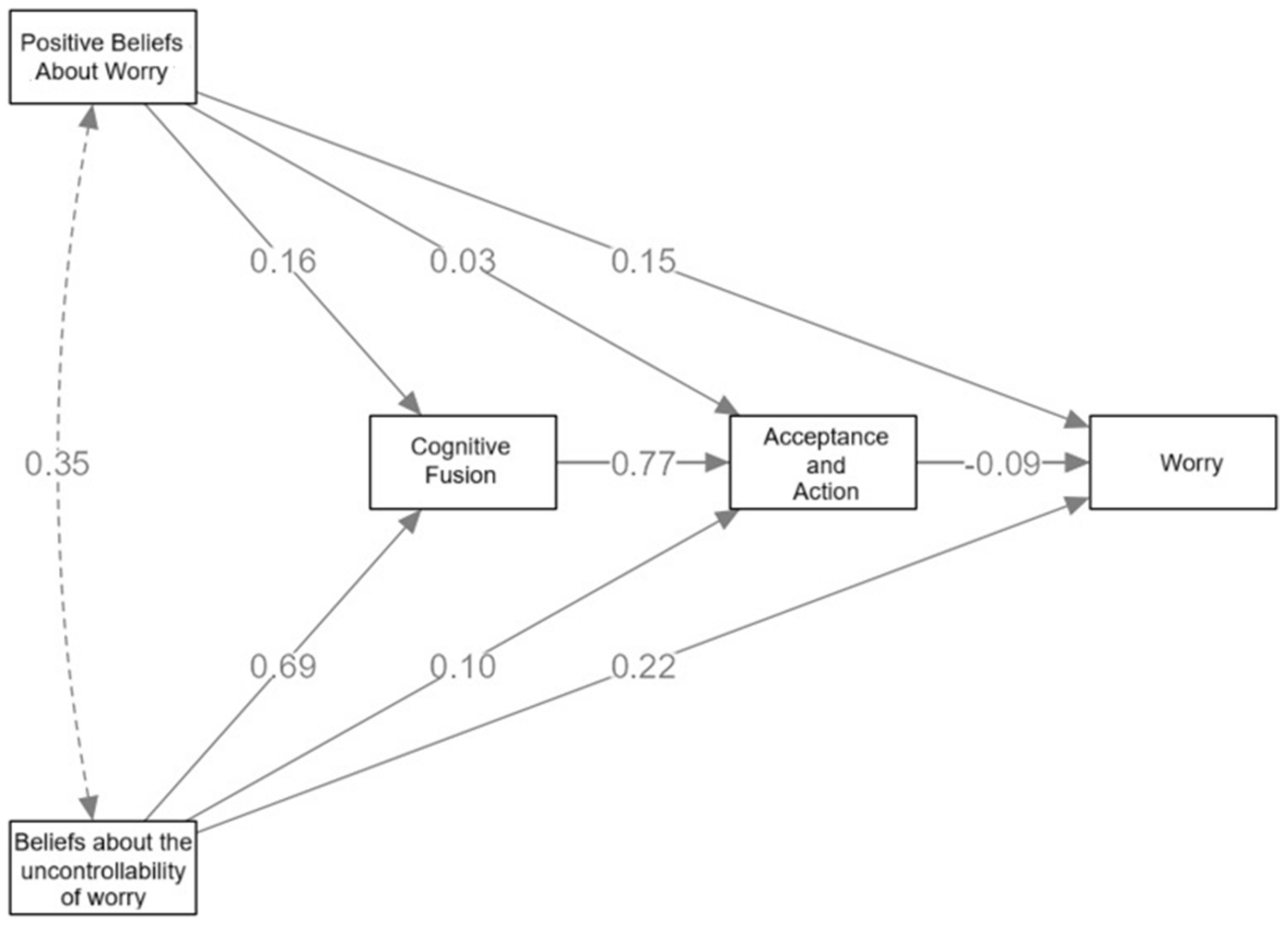
Mediation model linking metacognitive beliefs, cognitive fusion, acceptance and worry.

The mediation analysis described here (see Figure 1) reveals that cognitive fusion (measured by the CFQ) acts as a mediating variable between beliefs about the uncontrollability of worry (measured by the MCQ-30) and levels of acceptance (measured by the AAQ-II), as well as worrying (measured by the PSWQ). Metacognitive beliefs about uncontrollability are also directly related to worrying, which reflects the influence that such beliefs have on the persistence of the process of pathological worrying. The analysis indicates that both uncontrollability and metacognitive beliefs have direct and in-direct impacts on worrying, involving cognitive fusion and acceptance.

Specifically, beliefs about the uncontrollability of worry have a strong direct effect on cognitive fusion (*β* = 0.69), suggesting that individuals who believe they cannot control their worry are more likely to become entangled with their thoughts. Cognitive fusion, in turn, has a strong predictive relationship with reduced acceptance (*β* = 0.77), and a moderate effect on worry (*β* = 0.22). Although the direct path from acceptance to worry is small and negative (*β* = −0.09), it suggests that greater psychological flexibility (higher acceptance) slightly reduces worry levels.

Positive beliefs about worry show weaker direct relationships with fusion (*β* = 0.16), acceptance (*β* = 0.15), and worry (*β* = 0.03), but their overall contribution to the model may be indirect. The correlation between positive beliefs and beliefs about un-controllability (*β* = 0.35) indicates that these metacognitive dimensions are interrelated and may reinforce each other.

Table 3 presents the indirect, concurrent, and direct effects of the various variables involved in the model. The results show that the most significant indirect effects are those that follow the path of uncontrollability, cognitive fusion, and worrying (*β* = 0.9281, *p* < 0.001), thereby reinforcing the notion that cognitive fusion is a crucial mechanism in the relationship between beliefs about uncontrollability and high levels of worry. However, the 95% confidence interval for this effect was relatively wide (0.53–1.33), indicating limited precision in the estimation of its magnitude. This should be taken into account when interpreting the strength of this association.

**Table 3:** Indirect and total effects.

Type	Effect	Estimate	SE	95% CI (a)		β	z	*p*
				Lower	Upper			
Indirect	MCQ30 Uncontrollability ⇒ AAQII ⇒ PSWQ	−0.02066	0.0286	−0.0768	0.0355	−0.00875	−0.722	0.471
	MCQ30 Uncontrollability ⇒ CFQ ⇒ PSWQ	0.92814	0.2056	0.5252	1.3311	0.39308	4.514	<0.001
	MCQ30 Beliefs ⇒ AAQII ⇒ PSWQ	−0.00585	0.0119	−0.0291	0.0174	−0.00243	−0.492	0.623
	MCQ30 Beliefs ⇒ CFQ ⇒ PSWQ	0.21780	0.0904	0.0406	0.3950	0.09067	2.409	0.016
	MCQ30 Uncontrollability ⇒ CFQ ⇒ AAQII ⇒ PSWQ	−0.10844	0.1355	−0.3739	0.1570	−0.04593	−0.801	0.423
	MCQ30 Beliefs ⇒ CFQ ⇒ AAQII ⇒ PSWQ	−0.02545	0.0330	−0.0902	0.0393	−0.01059	−0.771	0.441
Component	MCQ30 Uncontrollability ⇒ AAQII	0.24835	0.1519	−0.0494	0.5461	0.10125	1.635	0.102
	AAQII ⇒ PSWQ	−0.08318	0.1034	−0.2859	0.1196	−0.08641	−0.804	0.421
	MCQ30 Uncontrollability ⇒ CFQ	1.83991	0.1531	1.5398	2.1400	0.68684	12.017	<0.001
	CFQ ⇒ PSWQ	0.50445	0.1036	0.3015	0.7074	0.57231	4.871	<0.001
	MCQ30 Beliefs ⇒ AAQII	0.07028	0.1129	−0.1511	0.2917	0.02816	0.622	0.534
	MCQ30 Beliefs ⇒ CFQ	0.43175	0.1558	0.1265	0.7370	0.15843	2.772	0.006
	CFQ ⇒ AAQII	0.70855	0.0579	0.5950	0.8221	0.77378	12.231	<0.001
Direct	MCQ30 Uncontrollability ⇒ PSWQ	0.50880	0.1936	0.1294	0.8882	0.21549	2.629	0.009
	MCQ30 Beliefs ⇒ PSWQ	0.37153	0.1428	0.0916	0.6514	0.15467	2.602	0.009
Total	MCQ30 Uncontrollability ⇒ PSWQ	1.30784	0.1536	1.0068	1.6088	0.55390	8.516	<0.001
	MCQ30 Beliefs ⇒ PSWQ	0.55804	0.1562	0.2518	0.8642	0.23232	3.572	<0.001

Confidence interval (CI) computed according to the standard Delta method. Betas are fully standardized effect sizes. AAQ-II, The Acceptance and Action Questionnaire – version 2; CFQ, The Cognitive Fusion Questionnaire; VQ, The Valuing Questionnaire; MCQ-30, Metacognitions Questionnaire-30; TCQ, Thought Control Questionnaire.

Furthermore, the concurrent effects reveal that uncontrollability has a considerable impact on cognitive fusion (*β* = 0.686, *p* < 0.001) and this, in turn, has a major impact on acceptance (*β* = 0.773, *p* < 0.001), which suggests that those individuals experiencing stronger beliefs about uncontrollability tend to merge more with their thoughts and show less acceptance of their internal experiences.

The direct effects are also significant, especially the direct paths from uncontrollability to worrying (*β* = 0.508, *p* = 0.009) and from beliefs about uncontrollability to worrying (*β* = 0.371, *p* = 0.009), which suggest that although cognitive fusion and acceptance mediate part of the relationship, beliefs about uncontrollability have a direct impact on worrying.

### Sensitivity analysis

To examine the robustness of the mediation model, we conducted a sensitivity analysis by including age, sex, and recruitment method (university vs. social media) as covariates. The indirect effect from metacognitive beliefs about uncontrollability to worry via cognitive fusion remained significant (*β* = 0.36, *p* < 0.001), and the overall structure of the model was preserved. Although age showed a small but significant negative effect on worry (*β* = −0.14, *p* = 0.049), neither sex nor recruitment group had a meaningful impact on the mediators or outcome. These results suggest that the main findings are not substantially influenced by demographic or sampling differences, strengthening the internal validity of the conclusions.

## Discussion and conclusions

This study aims to explore how key variables from metacognitive therapy and acceptance and commitment therapy can be integrated, with the aim of identifying overlapping processes that could inform future integrative approaches.

Our findings suggest that metacognitive beliefs about the uncontrollability of thoughts and positive beliefs about worrying are strongly associated with worry levels, primarily through their influence on cognitive fusion. This is consistent with metacognitive theory (Wells, 2009), which posits that negative beliefs about uncontrollability contribute to a self-perpetuating cycle of excessive worry and cognitive avoidance. Recent studies have further emphasized the central role of such beliefs in maintaining repetitive negative thinking (RNT) (Xu and Tsai, 2025; Kornacka et al., 2025). Cognitive fusion, a core ACT process, also emerged as a significant mediator, supporting previous evidence that identification with distressing thoughts amplifies emotional distress and worry (Hayes et al., 2006; Solem et al., 2011).

The results suggest that metacognitive beliefs about the uncontrollability of thoughts and beliefs about worrying have a considerable effect on worry, and carry the greatest weight, even though cognitive fusion has a highly significant impact on that relationship. According to Wells et al. (2009), uncontrollability contributes to the cycle of excessive worry by generating cognitive avoidance responses. The more one avoids worry-related thoughts, the easier it is to give them importance, and therefore trigger a cognitive fusion process (Solem et al., 2011). Cognitive fusion also mediates the relationship between positive beliefs about worrying and worry itself; in other words, believing that worry is beneficial may lead to increased focus on it, thereby intensifying distress (Wells, 2006). Although rumination was not measured in this study, future research could explore whether similar mechanisms apply across other forms of repetitive negative thinking, such as rumination.

In turn, cognitive fusion is directly related to the acceptance of thoughts and emotions - a central process in ACT. The inability to accept unwanted thoughts seems to reinforce cognitive fusion, which leads to an increase in levels of worry. This relationship between fusion and acceptance highlights ACT’s therapeutic value, which seeks to dismantle cognitive fusion by promoting experiential acceptance (Hayes et al., 2012). Moreover, this research shows that acceptance acts as a crucial mediator between cognitive fusion and worrying, which supports the hypothesis that the development of psychological flexibility through acceptance may alleviate worry and its associated emotional distress (Ruiz, 2010).

The mediation model also reveals that the weight of acceptance and action is significant in the prediction of worrying, which is consistent with other studies (Rood et al., 2012). Beliefs about the uncontrollability of worry may represent an important pathway to worry through their association with cognitive fusion, whereas acceptance appears to exert a smaller direct effect in this model (Wald and Mellenbergh, 2018). However, given that acceptance and cognitive fusion show similar zero-order correlations with worry, future research should examine whether their respective roles differ depending on contextual and population-specific factors.

Taken together, these findings tentatively suggest that future research could focus on evaluating whether interventions that help individuals accept the uncontrollability of thoughts are effective in reducing repetitive negative thinking.

This study presents several limitations that should be considered when interpreting the results. First, the cross-sectional and non-experimental design precludes any inference of causality among the variables examined. Although the tested mediation model is supported by theoretical frameworks, the directionality of the observed associations remains uncertain. To strengthen causal interpretations, future research should employ longitudinal or experimental designs that allow for the examination of temporal dynamics and causal pathways.

Second, the generalizability of the results may be limited by the characteristics of the sample. The sample was predominantly composed of female participants, which may restrict the applicability of the findings to the general population or to male sub-groups. Moreover, participants were drawn from a non-clinical population, and no formal diagnostic criteria were applied, which may further limit the extent to which these results can be extended to clinical settings or individuals with diagnosed anxiety or depressive disorders. Future research should aim to include more diverse and representative samples in terms of gender, age, and clinical status to enhance the external validity of the findings.

In short, the present findings highlight the central role of metacognitive beliefs—particularly those concerning the uncontrollability of thoughts—in sustaining worry. This effect appears to be primarily mediated by cognitive fusion, with acceptance contributing as a secondary mediator. These results align with prior evidence emphasizing the interplay between metacognitive processes and psychological flexibility (Hayes et al., 2006; Wells, 2009; Tamm et al., 2024).

From a clinical perspective, these insights suggest that interventions targeting both maladaptive metacognitive beliefs and experiential avoidance may offer complementary benefits. For instance, modifying beliefs about the uncontrollability of thoughts through detached mindfulness (MCT) (Mansueto et al., 2024; Wunder and Jones, 2023; Nordahl et al., 2022) could be combined with ACT strategies that promote acceptance and cognitive defusion (Cookson et al., 2020), which emphasizes the importance of fostering acceptance to reduce experiential avoidance and disengagement from unhelpful thought patterns (Hayes et al., 2006; Tamm et al., 2024). However, it is important to interpret these implications cautiously. Given the cross-sectional and exploratory nature of this study, causal relationships cannot be established. Further longitudinal and experimental research is needed to confirm these mechanisms and evaluate their relevance for integrative therapeutic approaches.

Future studies should also consider using more diverse and clinically representative samples, as the present study involved a predominantly female, non-clinical population. Moreover, replication in larger samples and across different cultural contexts would strengthen the generalizability of these findings. Designing experimental paradigms that assess worry responses in real-time (e.g., via ecological momentary assessment or laboratory-based induction tasks) could help clarify how metacognitive beliefs and cognitive fusion interact dynamically during episodes of repetitive negative thinking. Finally, some of the indirect effects observed in the model were associated with wide confidence intervals, suggesting reduced precision in estimating the magnitude of these effects. This limitation should be taken into account when interpreting the strength and reliability of specific pathways in the model.

Finally, future directions should also explore the integration of Metacognitive Therapy (MCT) and Acceptance and Commitment Therapy (ACT) techniques in ap-plied settings. Experimental studies testing the efficacy of interventions that combine strategies from both models—such as cognitive defusion exercises from ACT and attention training or detached mindfulness from MCT—could provide valuable in-sights into their synergistic effects. In addition, designing experimental studies that employ computer-based tasks to simulate worry-inducing scenarios could help assess how metacognitive beliefs and cognitive fusion processes influence worry responses in real time. These paradigms could offer an ecologically valid method to examine the immediate effects of interventions targeting fusion or metacognitive beliefs under con-trolled conditions. Furthermore, longitudinal models could be used to track the evolution of cognitive fusion, metacognitive beliefs, and acceptance over time, and their role in the onset and maintenance of pathological worry, allowing for a more comprehensive understanding of these transdiagnostic processes.

## Data Availability

The raw data supporting the conclusions of this article will be made available by the authors, without undue reservation.
