# Supplementary material for: Metabeliefs about worry, cognitive fusion, and acceptance: associations and mediations analysis
Source: Front Psychol. 2025 Aug 20;16:1639105. doi: 10.3389/fpsyg.2025.1639105 (PMC12405283; doi:10.3389/fpsyg.2025.1639105)
Supplement: Supplementary file 1 [file Supplementary_file_1.pdf]

Supplementary Material

Supplementary Results: Alternative Mediation Model

To examine potential alternative directional effects among key variables, we tested a mediation model in which cognitive fusion (CFQ) predicted worry (PSWQ), which in turn influenced acceptance (AAQ-II). In this model, MCQ-30 Uncontrollability and MCQ-30 Positive Beliefs were entered as predictors of cognitive fusion. Results showed that:
- MCQ-30 Uncontrollability had a strong direct effect on cognitive fusion ($\beta = 0.69$) and a smaller effect on worry ($\beta = 0.23$).
- Cognitive fusion strongly predicted acceptance ($\beta = 0.80$) and had a weak effect on worry ($\beta = 0.04$).

The indirect effect from MCQ-30 Uncontrollability to acceptance via cognitive fusion was significant ($\beta = 1.3462$, $p < .001$). However, the indirect effect from MCQ-30 Uncontrollability to acceptance through both cognitive fusion and worry was non-significant ($\beta = 0.086$, $p = .430$). MCQ-30 Positive Beliefs showed a significant indirect effect on acceptance via cognitive fusion ($\beta = 0.3159$, $p = .005$).

Overall, the alternative model yielded a comparable fit to the original one and provides insight into the potential bidirectional influences among cognitive fusion, worry, and acceptance. Nonetheless, the original model was retained in the main analysis due to stronger and more theoretically consistent predictive pathways.

Supplementary Figure S1

Alternative mediation model testing the pathway in which cognitive fusion (CFQ) predicts worry (PSWQ), which subsequently affects acceptance (AAQ-II). Metacognitive beliefs (MCQ-30 Uncontrollability and Positive Beliefs) were entered as predictors of cognitive fusion. Path coefficients are standardized.

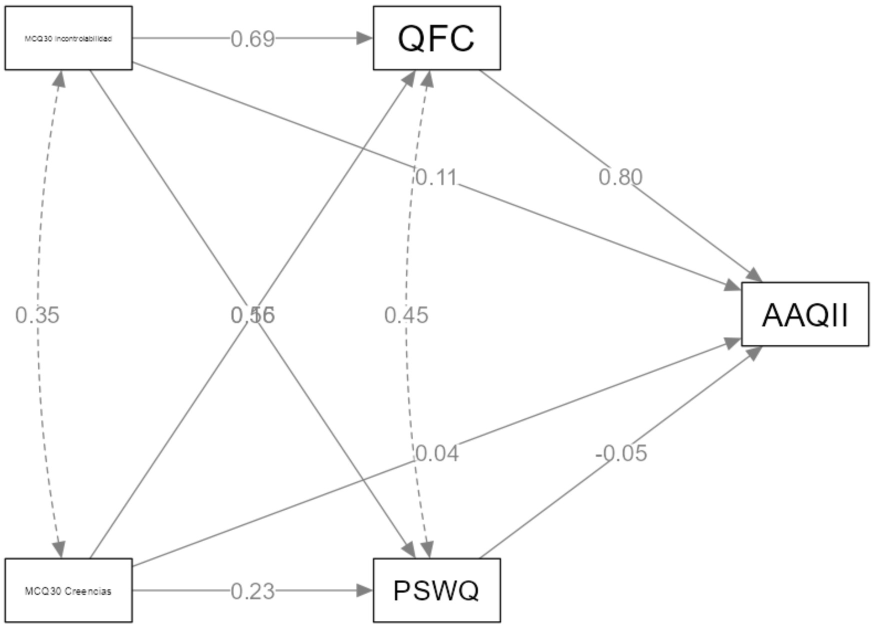

Supplementary Table S1: Indirect, Direct, and Total Effects

Indirect and Total Effects

Type	Effect	Estimate	SE	95% C.I. (a) Lower	Upper	β	z	p
Indirect	MCQ30 Creencias ⇒ PSWQ ⇒ AAQII	-0.0290	0.0399	-0.1044	0.0519	-0.0116	-0.727	0.467
	MCQ30 Creencias ⇒ QFC ⇒ AAQII	0.3159	0.1129	0.0895	0.5322	0.1266	2.798	0.005
	MCQ30 Incontrolabilidad ⇒ PSWQ ⇒ AAQII	-0.0679	0.0860	-0.2304	0.1068	-0.0277	-0.790	0.430
	MCQ30 Incontrolabilidad ⇒ QFC ⇒ AAQII	1.3462	0.1675	1.0184	1.6750	0.5488	8.037	< .001
Component	MCQ30 Creencias ⇒ PSWQ	0.5580	0.1462	0.2658	0.8387	0.2323	3.818	< .001
	PSWQ ⇒ AAQII	-0.0519	0.0653	-0.1757	0.0805	-0.0500	-0.795	0.427

Indirect and Total Effects

Type	Effect	Estimate	SE	95% C.I. (a) Lower	95% C.I. (a) Upper	β	z	p
	MCQ30 Creencias ⇒ QFC	0.4318	0.1479	0.1351	0.7150	0.1584	2.918	0.004
	QFC ⇒ AAQII	0.7317	0.0742	0.5859	0.8767	0.7990	9.861	< .001
	MCQ30 Incontrolabilidad ⇒ PSWQ	1.3078	0.1361	1.0329	1.5663	0.5539	9.611	< .001
	MCQ30 Incontrolabilidad ⇒ QFC	1.8399	0.1384	1.5701	2.1128	0.6868	13.290	< .001
Direct	MCQ30 Creencias ⇒ AAQII	0.0893	0.1441	-0.1921	0.3727	0.0358	0.620	0.535
	MCQ30 Incontrolabilidad ⇒ AAQII	0.2737	0.1653	-0.0478	0.6002	0.1116	1.656	0.098
Total	MCQ30 Creencias ⇒ AAQII	0.3762	0.1462	0.0844	0.6573	0.1508	2.574	0.010
	MCQ30 Incontrolabilidad ⇒ AAQII	1.5520	0.1514	1.2643	1.8577	0.6327	10.253	< .001

Nota. Confidence intervals computed with method: Parametric bootstrap

Nota. Betas are completely standardized effect sizes
